# Brain-Computer Interface Based on Generation of Visual Images

**DOI:** 10.1371/journal.pone.0020674

**Published:** 2011-06-10

**Authors:** Pavel Bobrov, Alexander Frolov, Charles Cantor, Irina Fedulova, Mikhail Bakhnyan, Alexander Zhavoronkov

**Affiliations:** 1 Institute of Higher Nervous Activity and Neurophysiology of Russian Academy of Sciences, Moscow, Russia; 2 Technical University of Ostrava, Ostrava Poruba, Czech Republic; 3 Department of Biomedical Engineering, Boston University, Boston, Massachusetts, United States of America; 4 Department of Physiology and Biophysics, University of California Irvine, Irvine, California, United States of America; 5 Moscow State University, Department of Computational Mathematics and Cybernetics, Moscow, Russia; 6 Moscow State University, Department of Physics, Moscow, Russia; 7 The Russian State Medical University, Moscow, Russia; University of Glasgow, United Kingdom

## Abstract

This paper examines the task of recognizing EEG patterns that correspond to performing three mental tasks: relaxation and imagining of two types of pictures: faces and houses. The experiments were performed using two EEG headsets: BrainProducts ActiCap and Emotiv EPOC. The Emotiv headset becomes widely used in consumer BCI application allowing for conducting large-scale EEG experiments in the future. Since classification accuracy significantly exceeded the level of random classification during the first three days of the experiment with EPOC headset, a control experiment was performed on the fourth day using ActiCap. The control experiment has shown that utilization of high-quality research equipment can enhance classification accuracy (up to 68% in some subjects) and that the accuracy is independent of the presence of EEG artifacts related to blinking and eye movement. This study also shows that computationally-inexpensive Bayesian classifier based on covariance matrix analysis yields similar classification accuracy in this problem as a more sophisticated Multi-class Common Spatial Patterns (MCSP) classifier.

## Introduction

A brain-computer interface (BCI) establishes a direct functional interaction between a human or animal brain and an external device. There are numerous recent advances in BCI development and implementation driven by scientific and technological achievements, as well as social and commercial demands.

Basic research has revealed correlations between brain signals and mental states [1], [2], [3], [4], [5]). This provides a variety of brain signals which might be used for BCI design [1]. Recent technological advances allow real-time on-line processing of multi-channel EEG data using low-cost commercial EEG devices (e.g. Emotiv EPOC EEG headset [6]). The proliferation of these devices into the consumer market has been accelerated by the ability to utilize BCI to partially restore function in various disabilities (see [2], [7]) and by a growing interest in using BCI for gaming and other consumer applications [8], [9], [10].

**Figure 1:**
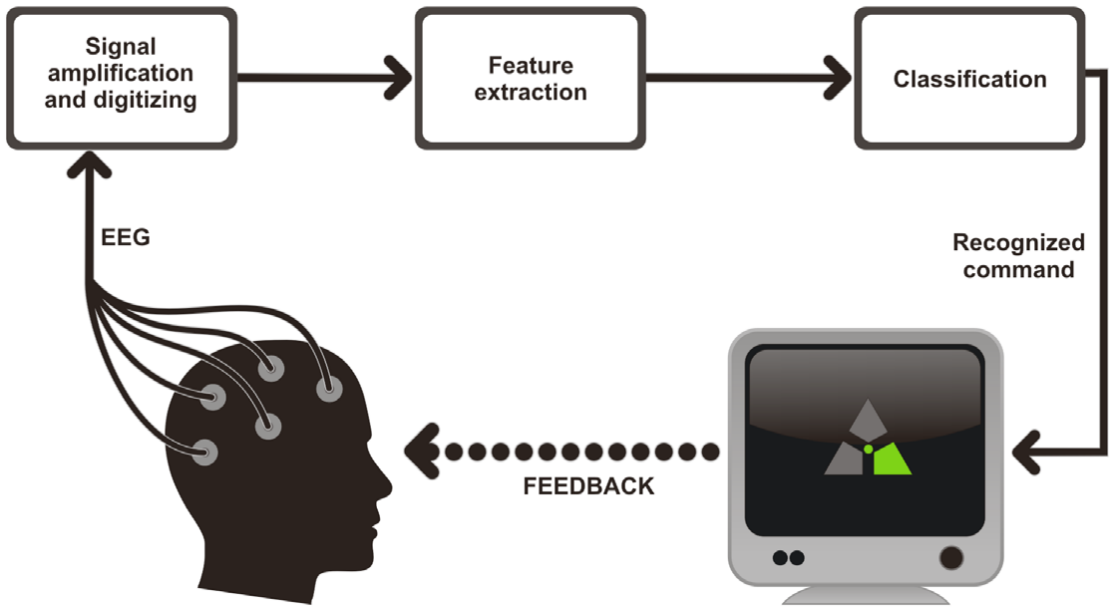
General scheme of an EEG-based BCI. EEG is recorded by electrodes placed on the scalp and digitized by an ADC. Computer processing extracts features most suitable for identifying the subject's intensions. When intension is classified, a certain command is sent to an external device (e.g., a display). Feedback provides the subject with results of his actions thus allowing him to adapt to the system behavior.

Figure 1 depicts a general scheme of an EEG-based BCI. The interface consists of an EEG acquisition system, data processing software for feature extraction and pattern classification, and a system to transfer commands to an external device and, thus, providing feedback to an operator.

One approach for BCI design is based on the discrimination of EEG patterns related to different mental states [4], [11], [12]. In this approach the subject is requested to perform different mental tasks. The classifier is trained to distinguish between EEG patterns related to these tasks. Execution of each task causes a certain command being sent to an external device, allowing the operator to control it by voluntarily switching between different mental tasks.

If commands sent to the external device trigger different movements, then psychologically compatible mental states are imaginary movements of different extremities. For example, when a subject controls a vehicle or a wheelchair, he can easily associate right hand movement with a right turn of the device. Moreover, mental states related to imaginary movements of extremities are clearly identified by corresponding EEG patterns (synchronization and desynchronization reactions of the mu rhythm, [13], [14]), as demonstrated in successful BCI projects such as Graz [2], [5], [15] and Berlin [16] BCI.

Potential applications of BCI extend beyond motion control, including controlling home appliances, selecting contacts in a phone address book or web search engine manipulation. Such tasks are more naturally accomplished by controlling the BCI with voluntary generation of corresponding visual images. Recent work by Cerf et al. [17] demonstrates human ability to voluntarily regulate the activity of neurons responsible for generation of visual images, however, their experiments were based on invasive recordings. As with motion imagination [18], [19], functional MRI data suggest that various spatial brain activation patterns correlate with specific types of imagined and perceived visual images [11], [20]. According to this data, generation of visual images activates nearly the same brain centers as does the perception of the actual image [21]. It has also been demonstrated that brain activity patterns vary not only by the type of the visual images, but also among images of the same type, and that analysis of such patterns allows to identify the image viewed by the subject, not only its type [22], [23]. These findings provide the rationale to hypothesize that brain activity patterns corresponding to specific generated visual images can be identified using EEG. Main goal of this study is to evaluate this hypothesis. In particular, it evaluates the opportunity to classify EEG patterns related to imagination of faces and houses. These types of generated visual images were shown to have different brain activity patterns in functional MRI studies [21], [24], [25].

One crucial part of a BCI system is the EEG pattern classifier, which identifies patterns corresponding to the subject's various mental states. There are many approaches for design of such classifiers [26]. The Common Spatial Patterns (CSP) method [27], allowing classification of states of two classes, and its multi-class generalization, the Multiple-class Common Spatial Patterns (MCSP) method [28], [29], [30] are widely used and considered to be quite efficient. This study compares the Bayesian and MCSP classifiers both based on EEG covariance matrix analysis. However, the Bayesian classifier has lower computational complexity than MCSP which makes it real-time adaptable.

An additional objective of this study is to evaluate the significance of EEG artifacts caused by blinking and eye movements, which generate patterns that can differ significantly in various mental states. Recognition of these artifacts can substantially improve EEG-based classification of these mental states. Patterns of involuntary eye movement may differ significantly, especially when different images are being imagined. Therefore, identification and removal of these artifacts is essential to ensure that BCI performance is based on classification of patterns of brain activity itself, and not based on eye-movement patterns.

This study was performed using two types of encephalographic caps: an easy to use readily available 16-channel EPOC (Emotiv Systems Inc., San Francisco, USA) and a 32-channel ActiCap (Brain Products, Munich, Germany). Emotiv EPOC is one of the most accurate consumer EEG headsets with the largest user community. This device can be potentially used to build a larger brain activity profile database by building a system for conducting remote experiments via web and opening it to the large user community. The experimental results were validated using ActiCap research EEG device since the research community is not yet actively using EPOC with few publications referencing the use of the device [10].

## Method

### Subjects

Seven male subjects aged from 23 to 30 participated in the study. All subjects were right-handed and had normal vision. The experimental procedure was approved by the Board of Ethics at the Institute for Higher Nervous Activity and Neurophysiology of the Russian Academy of Sciences. All participants signed the informed consent forms before participating in the experiments. All experiments involved non-invasive safe procedures and resembled a computer survey while using the non-invasive commercially-available EEG devices. The procedures were also described in the recruitment phase, where students and staff of several academic institutions were offered to participate in experiments involving EEG BCI.

### Experimental Design

**Figure 2:**
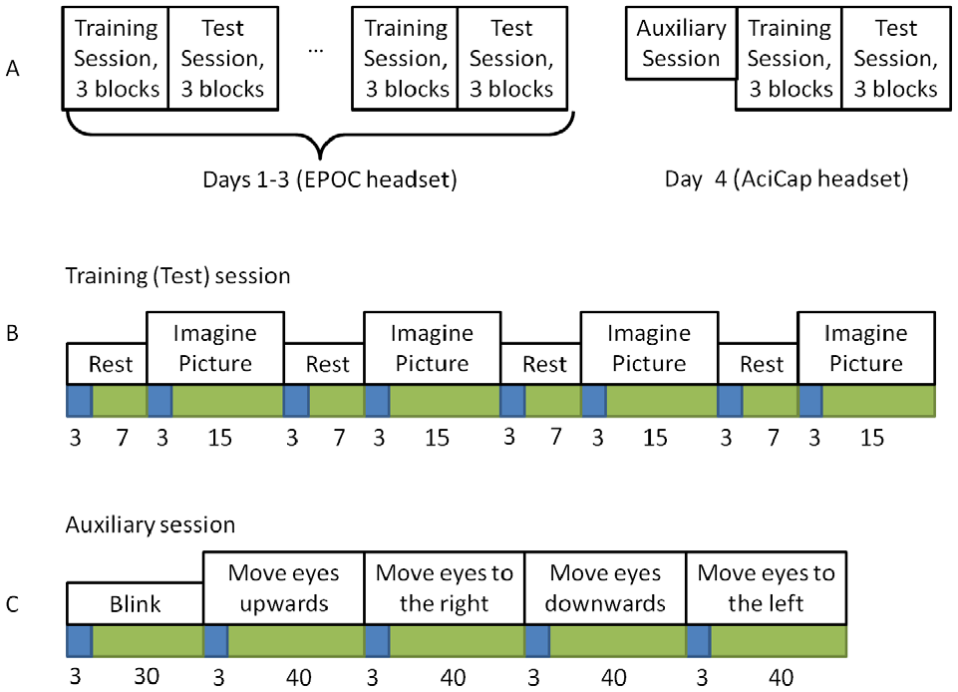
Schematic illustration of experiment protocol and each session timing. Sequence of sessions (A), structure of each training (test) session block (B), and structure of auxiliary session (C) are presented. Warnings are marked by blue and instructions to execute each task are marked by green. Instruction durations are given in seconds. Within each block, the instructions to imagine the face or the house are placed in random order, and each instruction is presented twice in a block.

The experimental protocol is schematically illustrated in Figure 2. The experiment was conducted on 4 consecutive days, the one series per day. Each series of the first three days consisted of two sessions (Figure 2A). The first, *training*, session was designed to train BCI classifier. The second, *test*, session was designed to provide subjects with the output of the BCI classifier in real time to enhance their efforts to imagine pictures. At the fourth experimental day the training and test sessions were preceded by *auxiliary* session, which was designed to obtain supplementary data for estimating the influence of EOG artifacts on the BCI performance. After the experiment was completed, the efficiencies of Bayesian and MCSP classifiers were compared offline. The influence of EOG artifacts on BCI performance was evaluated by comparing classification accuracy before and after the artifacts removal from the data of the fourth day.

At the beginning of the study each subject was presented with two types of pictures: faces (10 pictures from the Yale Face Database B [31]) and houses (10 pictures from the Microsoft Research Cambridge Object Recognition Data Base, version 1 [32], adjusted to black-and-white). Subject selected one face and one house as their preferred samples to imagine during the experiment.

Subject was sitting in a comfortable chair, one meter from a 17″ monitor. Subject was instructed to fix his gaze on a motionless circle (1 cm in diameter) in the middle of the screen, located at eye level. Three grey markers were placed around the circle as displayed in Figure 1. Green color of a particular marker indicated which mental task has to be performed. Left or right marker indicated that the subject should imagine a house or face. The top marker indicated relaxation. Each command to imagine a picture was displayed for 15 seconds and was preceded by a relaxation period of 7 seconds. Each clue was preceded by a 3-second warning (corresponding marker turned blue).

Each day experimental series had fixed correspondence of the marker and the picture. In the first series the left marker indicated face and the right marker indicated house. This relation was reversed in each sequential series to prevent classification based on steady marker position. Each series contained two sessions of three blocks (Figure 2(A)). The sessions were separated by a 5-minute interval. Within each block commands to imagine face or house were placed in random order, and each command was presented twice in a block (Figure 2(B)). Thus, each block displayed command to imagine a picture during 30 seconds and relaxation command during 28 seconds. In total, each picture has been imagined during 90 sec and subject has been relaxing for 84 sec in each of training and test sessions. The entire session took approximately 4.5 minutes. Before each session the subject had a chance to view and remember selected pictures.

Each day during the training session, the BCI classifier was trained to recognize three states: imagining the face, imagining the house, and relaxation. During the test session, the classifier was both trained and tested. The subject was provided with visual feedback: central circle turned green, if the classifier recognized the target state, otherwise it turned red. Each day the classifier was trained from scratch.

During the auxiliary session, the subject was asked to blink (approximately once per second) and to move gaze from the center of the screen in the indicated direction, and back, fixing gaze for 0.5 sec in each position. The directions were up, right, down, and left. Each eye movement condition took 40 seconds, and the blinking condition took 30 seconds (Figure 2(C)).

### Data recording

**Figure 3:**
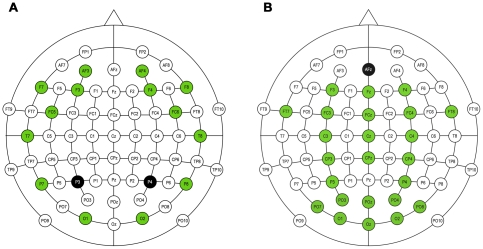
Electrode locations for EEG headsets used in this study. EPOC (A), ActiCap (B).

During the first three days of the study, EEG was recorded using the Emotiv Systems Inc. (San Francisco, USA) EPOC 16-electrode cap (Figure 3(A)). The electrodes were located at the positions AF3, F7, F3, FC5, T7, P7, O1, O2, P8, T8, FC6, F4, F8, AF4 according to the International 10–20 system. Two electrodes located just above the subject's ears (P3, P4) were used as reference. The data were digitized using the embedded 16-bit ADC with 128 Hz sampling frequency per channel and sent to the computer via Bluetooth. The data were band-pass filtered in 5–30 Hz range. The impedance of the electrode contact to the scalp was visually monitored using Emotiv Control Panel software.

On the 4^th^ day of the experiment, EEG and EOG (electrooculogram) were recorded using the Brain Products, (Munich, Germany) ActiCap (Figure 3(B)): 24 electrodes (Fz, F3, F4, Fcz, Fc3, Fc4, FT7, FT8, Cz, C3, C4, Cpz, Cp3, Cp4, P3, P4, Poz, Po3, Po4, Po7, Po8, Oz, O1, O2) were used to record EEG and 6 (SO1, IO1, LO1, SO2, IO2, LO2) electrodes, placed around the eyes, were used to record EOG. Central frontal electrode (Afz) was used as reference. The level of electrode impedances was evaluated by means provided by cap producers. The signals were displayed in real time on the computer screen that allowed for controlling their quality visually. The signal provided by EPOC seemed to be noisier than by ActiCap. This observation was confirmed quantitatively during the deleting of EEG artifacts.

The data were acquired with 200 Hz sampling frequency and band-pass filtered in 1–30 Hz range by a computer encephalograph (NBL640, NeuroBioLab, Russia) and additionally filtered in 5–30 Hz range by a software FIR filter using MATLAB Filter Design toolbox. All other data processing was also carried out with MATLAB (the Mathworks Inc., Natick, MA, USA).

### EEG pattern classification

The algorithms used for mental state classification are described in the following sections.

#### Bayesian approach

Suppose that there are 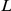 different classes of mental states and for each mental state the EEG data distribution is approximately Gaussian with zero mean. Assume that 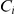, a covariance matrix of the data corresponding to the 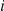-th mental state, is nonsingular. Then, the probability to obtain signal 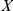 under the condition that it corresponds to the 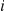-th mental state is 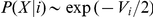, where 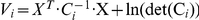. Following the Bayesian approach, the maximum value of 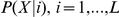, determines the class to which 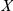 belongs. Hence, the signal 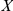 is considered to correspond to the 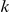-th mental state as soon as 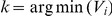. The equality 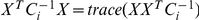 implies that
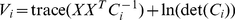
(1)Because all 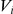 are rather variable, it is more beneficial to compute the mean values 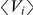 for sequential EEG epochs using (1)
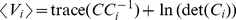
(2)where 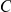 denotes an epoch data covariance matrix computed as 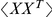.

Therefore, to perform the classifier learning it was sufficient to compute the covariance matrices corresponding to each mental state. The classifier was tested by approximating the covariance matrix for each 1-second EEG epoch and computing 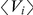 according to (2). In addition, during the test session the classifier was adjusted after the end of each block. For each mental state the covariance matrix 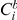 was computed based on the block data and the covariance matrix 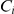 was replaced with 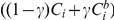, where the parameter 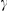 is 0.01.

#### MCSP method

This approach is based on covariance tensor analysis [30]. In case where tensors are second order (i.e., they are covariance matrices), the MCSP method can be described as follows. The covariance matrices 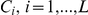 are obtained based on multi-dimensional EEG data recorded during the classifier learning. Then matrices 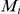 are sought to meet the following requirements
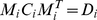
(3)

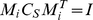
(4)where 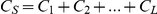, 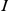 is the identity matrix, and 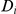 is a diagonal matrix. The problem of obtaining the matrices 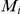 has an explicit solution. Indeed, if 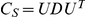 is the singular value decomposition (SVD) of the 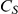 matrix, with 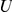 a unitary matrix and 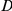 a diagonal matrix, then it is easy to prove that 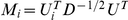, where 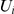 is the unitary matrix obtained from the SVD decomposition 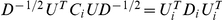. The matrices 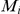 are used to project both the training and the test data onto feature space and, therefore, to obtain a set of training and test feature vectors for each state. The signal corresponding to a certain state is segmented into epochs and for each epoch vectors 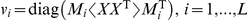, are computed by estimating variances of all components of vectors 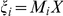 based on the epoch data 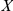. Then 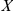 is mapped onto a feature vector 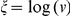, where 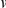 is concatenation of all vectors 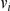, and 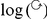 is a component-wise log-transform. After that, classification of the test feature vectors is performed. We used the SVM algorithm described in [33] for feature vector classification.

When two mental states are classified, results obtained by MCSP and CSP [27] are identical. In this case 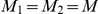 and 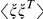 is a diagonal matrix. If a particular component of 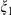 has low variance for a certain state, then this component of 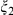 has high variance in this state and vice versa, since 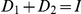. Then feature vectors corresponding to different states can be easily separated. Similarly, when MCSP is applied to classify more than two states, then for each state 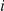 there exist components of 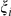 with variance significantly lower than variances of the respective components of 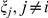. This can explain the efficacy of MCSP.

### Evaluation of classifier quality

To compare Bayesian and MCSP classifiers, offline analysis of both training and test session data was performed. The comparison was offline for several reasons. At first, the training session data could not be analyzed online, because the classifier had not been trained yet. Secondly, it was reasonable to use only one classifier (Bayesian in our experiments) for online feedback control of the mental states during the test session.

To evaluate classifier efficiency, EEG records corresponding to different mental states were split into epochs of 1 second length. Then the artifact data were identified according to the 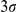 rule. All epochs with more than 7% of samples marked as artifact were excluded from the analysis. After the exclusion of the artifact epochs, repeated random sub-sampling validation was performed. The epochs were split into training and test sets with 90% of epochs being used for classifier training and 10% epochs being used for testing. Each learning set contained about 70 epochs and about 7 epochs were used for classifier testing in each of two sessions for EPOC and, respectively, about 75 and 8 for ActiCap. As a result of averaging over 100 splits, a confusion matrix 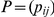 was obtained. Here 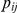 is an estimate of probability to recognize the 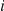-th mental state in case the 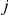-th mental state is to be produced. Note that in case of the good state recognition diagonal elements of matrix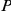 are significantly greater than non-diagonal ones; and if there is no classification error, then 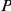 is identity matrix.

Mean of the confusion matrix diagonal elements
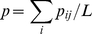
(5)was chosen as an index of the classification quality. It is easy to see, that 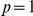 when the states are recognized perfectly, and 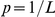 if classification is independent of the mental states produced.

The classifier performance was also measured by computing the mutual information between the commands to produce mental states and the states classified:
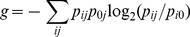
(6) In equation (6) 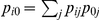 is probability of the 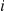-th state to be recognized and 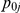 is probability of the *j*-th command to be presented. Notice that if probabilities to display each command are assumed to be equal, then 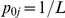. In this case 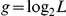 as soon as there is no classification error. Also notice that 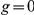 when the state recognition is independent of the commands.

Consider a special case when probabilities of correct recognition of different mental states equal to each other, i.e. 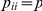 for all 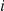, and probabilities of incorrect recognition also equal each other, i.e. 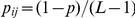 for all 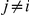. Then the mutual information between the displayed commands and the states classified can be obtained as follows:
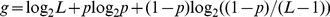
(7) Based on [34], equation (7) is often used to estimate BCI efficacy ([35], [36], [37]). But if the corresponding assumptions are not true, the value of 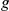, calculated according to (7), is lower than the actual mutual information. In this study we used the general formula (6).

### EOG artifact removal

To evaluate the influence of EOG artifacts on BCI performance we compared BCI efficiencies before and after artifact removal from recordings of the fourth day. To remove the artifacts from recordings of the training and test sessions we concatenated the recordings with those of the auxiliary session. These data were not filtered in 5–30 Hz range in order to avoid artifact attenuation that could impair their detection. To identify the artifacts we used implementation of the Infomax Independent Component Analysis (ICA) algorithm (EEGLAB RUNICA, [38]). As a result of ICA, multidimensional signal 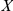 containing both EEG and EOG data, is represented as
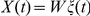
where 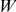 is a matrix of weights where columns specify contribution of the corresponding independent component into each EEG or EOG channel and vector 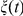 specifies intensities of the independent components. Since 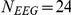 electrodes were used to record EEG and 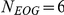 electrodes were used to record EOG, 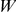 is a 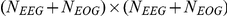 matrix. The obtained independent components were sorted according to their contribution to the total variance of the EOG signals. The first 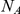 components constituting 97% of the variance were treated as artifact components. Artifact removal was performed by setting intensities of artifact components to zero. This is equivalent to removing the first 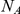 columns of *W*. Thus, refined EEG signal is represented as
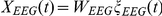
where 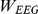 was a 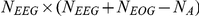 matrix of weights and 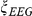 was 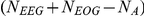-dimensional vector of non-artifact source intensities. The 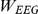 matrix was obtained from the 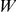 matrix by removing 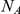 columns corresponding to the artifact components and 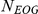 rows corresponding to the EOG channels.

## Results

The first part of this section demonstrates the results of the offline classifier comparison. We also show that the EOG input into EEG is sufficient for BCI control. This emphasizes importance of EOG artifact removal to obtain BCI based on brain activity only, and not based on eye movement and blinking. Afterwards, results of classification of the data with EOG artifacts removed are presented.

### BCI efficiency

Table 1 shows the confusion matrix obtained for one subject by offline Bayesian classification of the data recorded during the 4^th^ day training session. It can be seen that matrix is diagonally dominant, which means that correct recognition is prevalent. In this case 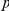, mean of the matrix diagonal elements, equals to 0.54 and 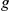, mutual information, equals to 0.14. In contrast, 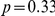 and 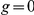 if three states were classified randomly.

**Table 1:** Confusion matrix obtained with the Bayesian classifier for one subject based on the data of the 4^th^ day learning session.

		Commands presented		
		Relax	Imagine the house	Imagine the face
Recognized states	Relaxation	0.53	0.15	0.18
	Imagining the house	0.20	0.52	0.24
	Imagining the face	0.27	0.33	0.58

**Figure 4:**
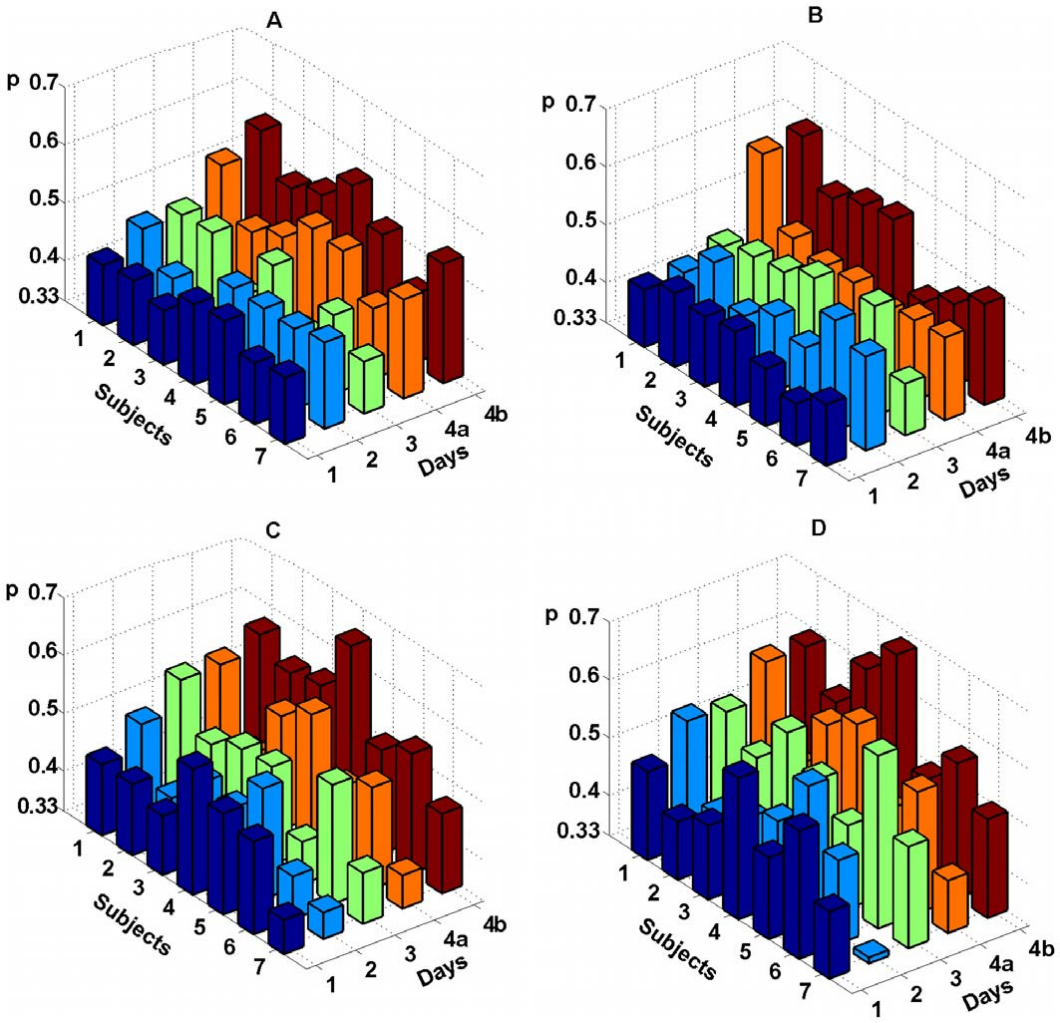
Classification quality for all subjects during training and test sessions, as measured by value 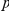. Classification quality during training is displayed on left panes A and C, quality during test is presented on panes B and D. The first row of columns corresponding to the 4^th^ day (4a) represents 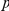 values computed from data for 16 EEG electrodes, and the second one (4b) represents the values computed from data for all 24 EEG electrodes. Notice that each column exceeds the level *p* = 0.33 related to random classifying.

**Figure 5:**
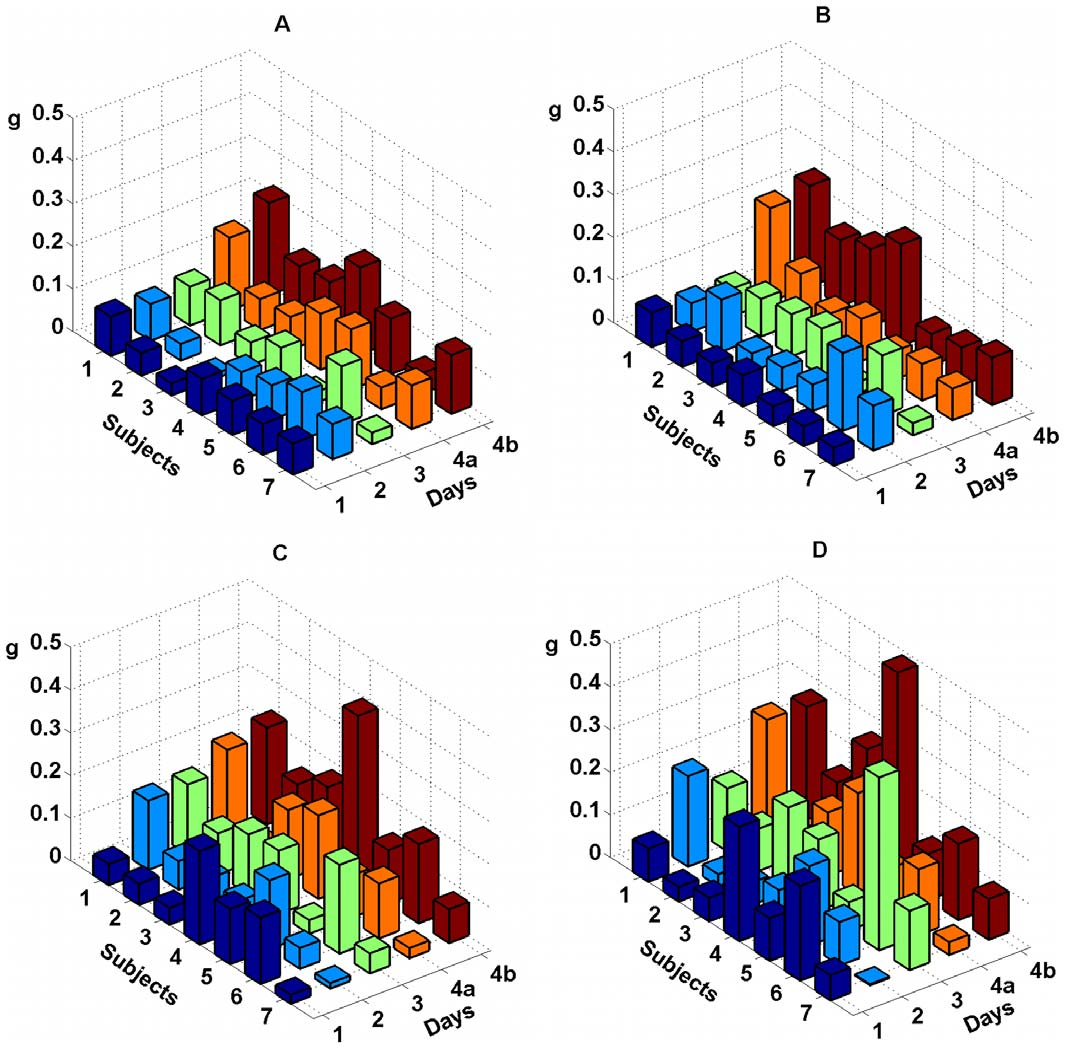
Quality of classification measured by index 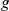. Data representation is the same as in Figure 4.

Figures 4 and 5 show 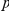 and 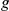 values for all 4 experimental days for all subjects. Left panes (A and C) of each figure represents classification quality for Bayesian approach and right panes (B and D) represent quality for MCSP. Respectively, upper panes (A and B) represent quality for training session and lower panes (C and D) represent quality for test session. For every subject and every session of each day values of 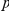 exceed the value of 0.33 which corresponds to random classification.

Two-way ANOVA test for EPOC data did not reveal dependence of 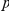 and 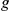 indices on the day of experiment (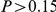 for both sessions, both classifiers, and both indices). However this test revealed significant increase of 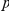 and 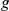 when comparing MCSP classification of training and test sessions (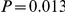 for 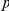 and 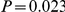 for 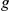). At the same time, the increase was not significant for Bayesian classification (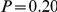 for 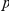 and 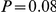 for 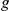). Two-sample t-test for ActiCap data revealed that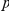 index was significantly higher for MSCP classification of the test session than for training session (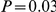). Observed trend of quality improvement in the test session might be explained by subjects' additional training during the preceding training session as well as the increase in subjects' focusing on the task when they were provided with visual feedback.

Also ActiCap data were classified significantly better than EPOC data (two-sample t-test, 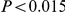 for both training and test sessions and both classifiers). For the ActiCap data the maximum value of 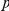 over all subjects equals to 0.66 (0.68) for the Bayesian (MCSP) classifier and the maximum value of 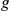 over all subjects equals to 0.39 (0.48) for the Bayesian (MCSP) classifier. Average values of 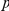 and 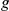 over all subjects and sessions equal 0.52 (0.56) and 0.15 (0.20) for Bayesian (MCSP) classifier. For the EPOC data maximum values over all subjects and experiment days equals to 0.52 (0.63) for 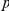 and 0.18 (0.40) for 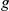. Average values of 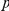 and 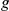 over all subjects, sessions, and days equals to 0.45 (0.48) and 0.07 (0.11) correspondingly. It can be seen that on average MCSP classifier based on covariance matrix analysis performed slightly better than the Bayesian one. The difference in classification quality was small but significant for EPOC data (two-sample t-test, 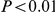 for 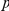 and 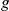 over all subjects, experimental days, and sessions) although not significant for ActiCap data (two-sample t-test, 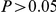 for 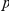 and 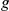 over all subjects and sessions).

### Influence of EOG artifacts

It could be possible that the relatively high BCI quality observed is related to eye movement. During imagining selected pictures subjects might be making involuntary eye movements in specific patterns, detailing the imagined pictures. Below we show that classification between states is actually based on differences in brain activity measured by EEG, and not on patterns of eye blinking and movements.

To investigate this we demonstrated that patterns resulting from eye movements and blinking on EEG could be easily discriminated by means of the proposed classifiers. On the fourth experimental day auxiliary session data were processed as described in *Evaluation of classifier quality* section above. Recall that the EEG patterns recognized were induced by blinking and eye movements of four types (up-center, right-center, down-center and left-center). Table 2 shows the confusion matrix coefficients (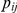) for one subject, obtained as a result of Bayesian classification. The diagonal coefficients of the matrix presented are observably dominant indicating high classification quality. Mean values of 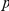 among the subjects were 0.63±0.04 and 0.64±0.04 for the Bayesian and the MCSP classifiers respectively, and corresponding mean values of *g* were 0.86±0.14 and 0.85±0.13. Note that 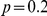 when five states are classified randomly. The mutual information 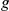 for the EEG patterns mentioned above is an order of magnitude greater than the quality achieved in recognition of patterns that correspond to imagining the pictures.

**Table 2:** Confusion matrix obtained as a result of Bayesian classification of EEG patterns, corresponding to various eye movements and blinking, prior to EOG artifact removal.

		Commands presented				
		Blink	Move gaze upwards	Move gaze right	Move gaze downwards	Move gaze left
Recognized states	Blinking	0.73	0.06	0.00	0.05	0.00
	Upward eye movements	0.04	0.66	0.02	0.10	0.01
	Rightward eye movements	0.00	0.04	0.81	0.05	0.27
	Downward eye movements	0.22	0.21	0.01	0.78	0.02
	Leftward eye movements	0.01	0.03	0.16	0.02	0.70

**Figure 6:**
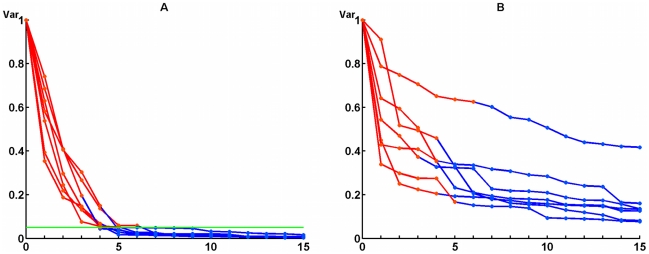
Decrease of total variance of signals after sequential removal of the independent components for all subjects. Left pane (A) represents signals recorded by EOG electrodes, and right pane (B) corresponds to EEG electrodes. Removed artifact components are marked by red points.

Next, EOG artifacts were removed from the auxiliary session data using the method described in section *EOG artifact removal*. Briefly, the ICA decomposition of the signal was obtained and the components comprising the 97% of total variance of signal from EOG channels were eliminated. Figure 6(A) shows residual values of EOG signal variance during the sequential removal of components rated according to their contribution. Results are shown for all 7 subjects. Margin of 3% is also shown in Figure 6(A). Note that the number of the artifact components never exceeded the number of EOG channels so the covariance matrices computed based on the refined data were never singular. Figure 6(B) shows decrease of the total variance of signals from EEG channels during the sequential removal of the EOG-related components. Contribution of the components into EEG signal is quite substantial, but their removal suppresses EEG signal less significantly than it does for EOG signal. Total EEG variance, averaged over all subjects, remains at 30% after artifacts are removed.

**Figure 7:**
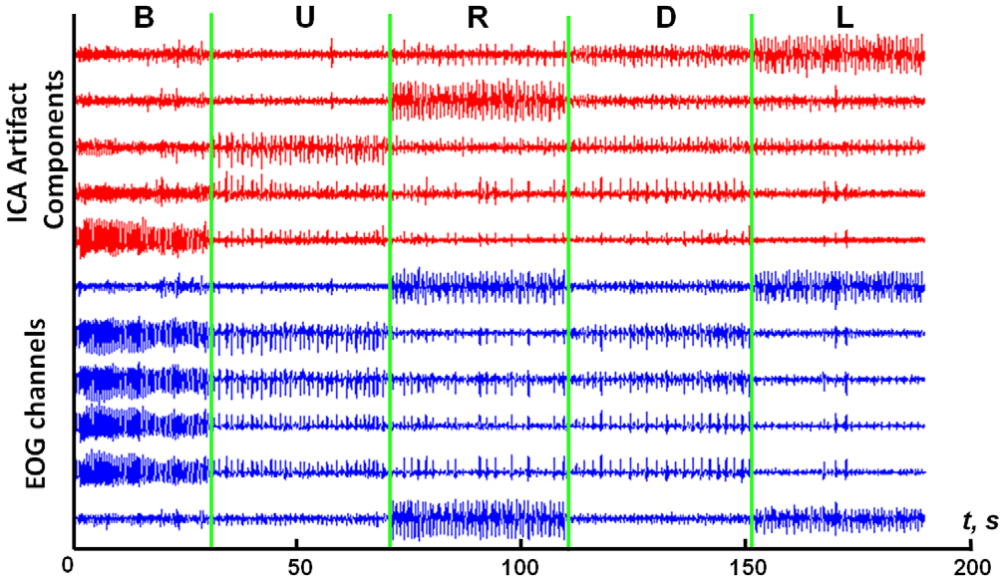
Signals from EOG electrodes for one of the subjects and corresponding independent components, identified as EOG-related for this subject. EOG signals are presented in blue (6 lower curves), corresponding independent components are displayed in red (5 upper curves). From left to right, signals correspond to: blinking (B), moving eyes upwards (U), to the right (R), downwards (D) and to the left (L). Each curve is normalized by the standard deviation.

**Figure 8:**
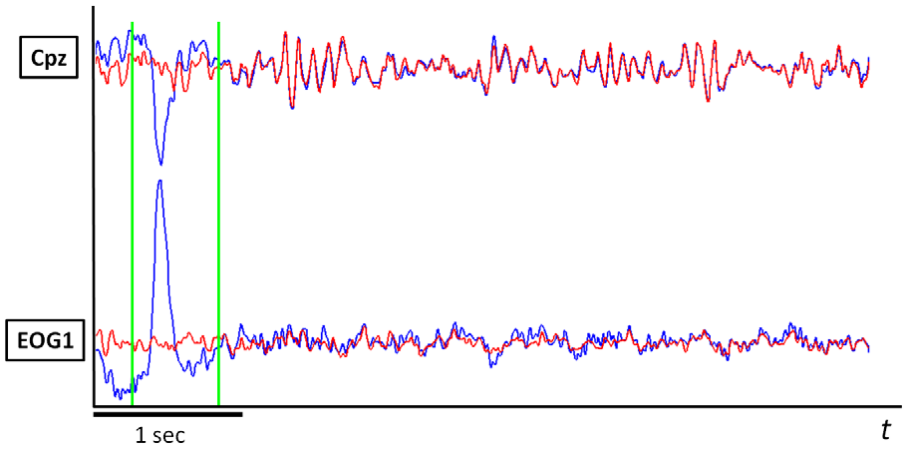
Result of blinking artifact suppression for one of the EOG channels and one of EEG channels. Blue and red curves represent signals before and after artifact components removal respectively.

Figure 7 shows the signals from 6 EOG electrodes for one subject and 5 independent components, identified as EOG-related for this subject. There is an evident correspondence between the components and types of EOG artifacts. Figure 8 demonstrates the result of EOG artifact removal. The data previously contaminated with artifacts became indistinguishable from the data initially containing no artifacts.

**Figure 9:**
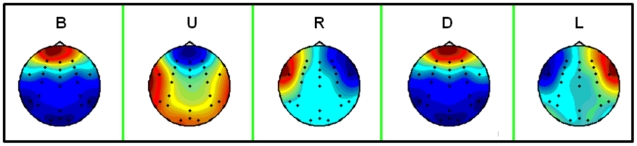
Spatial distributions of individual ICA components related to EOG artifacts. Graphs correspond to blinking (B), moving eyes upwards (U), to the right (R), downwards (D) and to the left (L).

Figure 9 presents distributions of individual ICA components related to EOG artifacts over the head. They agree with distributions for modeled blinking and eye movement artifacts, described in [39].

The confusion matrix for data obtained in auxiliary session of the fourth day after EOG artifact removal is presented in Table 3 for the same subject as in Table 2. As shown, recognition quality is substantially reduced due to artifact removal. Mean values of *p* among the subjects dropped to 0.50±0.03 and 0.42±0.03 for the Bayesian and MCSP classifiers respectively, and corresponding mean values of *g* dropped to 0.47±0.10 and 0.29±0.06. The difference of quality measures computed before and after artifact removal is significant (two-sample t-test, 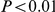 for both classifiers and both measures). It is remarkable that even with nearly complete exclusion of EOG artifacts the quality of EEG pattern classification remained quite high and significantly exceeded random level (one-sample t-test performed for 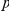 index, 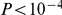).

**Table 3:** Confusion matrix obtained as a result of Bayesian classification of EEG patterns, corresponding to various eye movements and blinking, after EOG artifact removal.

		Commands presented				
		Blink	Move gaze upwards	Move gaze right	Move gaze downwards	Move gaze left
Recognized states	Blinking	0.49	0.22	0.04	0.02	0.12
	Upward eye movements	0.04	0.37	0.08	0.10	0.07
	Rightward eye movements	0.30	0.24	0.51	0.28	0.14
	Downward eye movements	0.01	0.10	0.20	0.39	0.23
	Leftward eye movements	0.16	0.07	0.17	0.21	0.44

The second step to investigate possible EOG artifact effect on classification of EEG patterns corresponding to imagining the pictures was to evaluate the quality after EOG artifact removal. Data obtained on the fourth experimental day were used since EOG was recorded only during this day. During processing training and test sessions, each session data was filtered in 1–30 Hz range and concatenated with the auxiliary session data. The concatenated records were decomposed using ICA to identify EOG-related components and eliminate them. The artifact components were quite similar to those obtained during processing the auxiliary session data alone. Their number was the same for each subject and they could be easily attributed to eye movements of a particular type or blinking. After EOG artifacts were removed the data auxiliary session records were discarded, the remaining EEG data were filtered in the 5–30 Hz range and used for off-line classification quality estimation.

The result of the classification is represented by Table 4. All changes in classification quality measured by 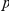 or 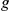 are insignificant for both classifiers and both training and test sessions (two-sample t-test, 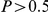 in all cases). This indicates that state classification is actually based on differences in brain activity measured by EEG, but not on patterns generated by eye blinking and movement.

**Table 4:** Comparison of EEG pattern recognition quality for the training and the test sessions, before and after EOG artifact removal.

		Training session		Test session	
		Bayesian	MCSP	Bayesian	MCSP
Artifacts included	p	0.52±0.02	0.51±0.02	0.55±0.02	0.57±0.03
	g	0.14±0.02	0.15±0.03	0.19±0.04	0.22±0.05
Artifacts excluded	p	0.51±0.01	0.51±0.02	0.54±0.02	0.57±0.02
	g	0.13±0.02	0.15±0.03	0.17±0.04	0.22±0.05

### Features of EEG patterns relevant for BCI performance

To reveal which features of EEG patterns are important for recognition of three considered mental tasks we found frequency bands and EEG electrodes most contributing to BCI performance. The data of the last fourth experimental day obtained by ActiCap for test session were chosen for analysis because these data were classified best.

The total frequency range was divided into 6 not overlapping frequency bands (5–7, 8–12, 13–17, 18–22, 23–26, 27–30 Hz) and BCI performance was evaluated by Bayesian classifier for each of the bands. The band 8–12 Hz happened to be most relevant. It provides the classification quality *p* = 0.55 that is equal to the quality obtained for the whole frequency range of 5–30 Hz (see Table 4). The same quality was achieved for any combination of bands containing 8–12 Hz band.

**Figure 10:**
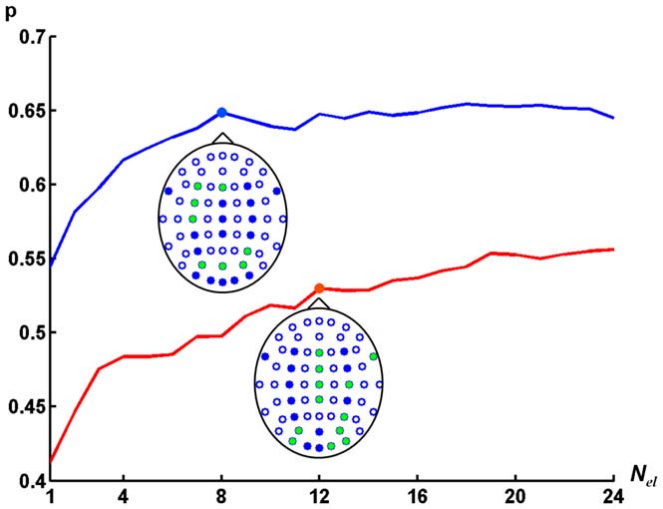
Classification quality *p* depending on the number of electrodes. Red graph corresponds to classification among three mental states (imagining of two pictures and relaxation). Blue graph represents classification of two mental states (imaging of two pictures, relaxation is not considered). Optimal electrode configurations are shown by green dots.

The quality *p* was also calculated in dependency on the number *N_el_* of electrodes used for EEG classification. To find the optimal configuration of electrodes of given number we used a “greedy” algorithm which discarded electrodes one by one starting from the set of all EEG electrodes. At each step an electrode was removed from the set of electrodes, obtained at the previous step, so that remaining set of electrodes provided the highest classification accuracy. The quality *p* in dependence on *N_el_* averaged over all subjects is shown in Figure 10. The quality monotonously decreases with reducing number of electrodes but the rate of decrease becomes larger when *N_el_* reaches 12. Therefore we treat that set of 12 electrodes as most relevant for EEG pattern classification. The locations of these electrodes at the head are shown in Figure 10. Notably, the electrodes P4, Po4, Po8, Po3, and Po7 are among 12 optimal electrodes. EEG recorded by these electrodes might reflect the activity of the areas in *medial fusiform gyri*, *lateral fusiform gyri* and *iferior temporal gyri* which are found to be related to imagination of faces and houses using fMRI study [21].

Although the confusion matrices for individual subjects have not revealed the dominance of one of the considered mental tasks (e.g. Table 1), analysis of optimal frequency band and optimal electrode configuration showed that posterior alpha rhythm is most relevant to BCI performance. Since alpha rhythm on posterior electrodes is believed to reflect the change in attention [40] one can expect that subjects' increased awareness during picture imagination compared to relaxation is the decisive factor underlying classification ability. That is why we analyzed the classifier ability to distinguish between each pair of mental tasks. The results are shown in Table 5. Despite the fact that relaxation is actually better distinguished from each picture imagination, the quality of two pictures classification is comparable and significantly exceed the level of random classification (one-sample t-test, *P*<0.001 for both sessions, classifiers and measures). Remind that the level of random classification of two classes amounts to *p* = 0.5.

**Table 5:** Results of pair wise EEG patterns recognition for the training and test sessions of the last experimental day.

		Training session		Test session	
		Bayesian	MCSP	Bayesian	MCSP
Relaxation vs. face	p	0.69±0.03	0.68±0.03	0.73±0.03	0.73±0.03
	g	0.12±0.03	0.12±0.03	0.18±0.04	0.19±0.06
Relaxation vs. house	p	0.72±0.02	0.70±0.02	0.70±0.03	0.70±0.03
	g	0.15±0.03	0.14±0.03	0.15±0.04	0.14±0.05
House vs. face	p	0.62±0.01	0.63±0.02	0.64±0.02	0.64±0.02
	g	0.05±0.01	0.05±0.01	0.07±0.01	0.07±0.02

The quality *p* of classification of two pictures in dependence on number of electrodes is also shown in Figure 10. The quality does not decrease until the number of electrodes is *N_el_* = 8. We treated the remaining electrodes as most relevant for classification of EEG patterns corresponding to imagination of faces and houses. The found optimal configuration of 8 electrodes is shown in Figure 10. Remarkably, the configuration contains P4, Po3, and Po4 electrodes as for classification of 3 mental tasks.

## Discussion

This study demonstrates that patterns of brain activity formed by imagining pictures can be classified based on EEG recordings obtained by both used headsets: Emotiv EPOC and BrainProducts ActiCap. The percentage of correctly recognized states significantly exceeded 33%, which is percentage of the states classified at random. It averaged 48% for the EPOC data and 54% for the ActiCap data, while for some subjects it was as high as 62% (EPOC) and 68% (ActiCap). The classification quality evaluated by information measure 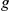 averaged 0.11 bit/sec (6.6 bit/min, EPOC) and 0.17 bit/sec (10.2 bit/min, ActiCap), approaching 0.40 bit/sec (24 bit/min, EPOC) and 0.48 bit/sec (29 bit/min, ActiCap) for some subjects. These results are comparable to characteristics of BCIs based on motion imagination. For example, the BCI described by [16], had average 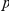 equal to 88% when two states were recognized, and its information index of classification quality averaged 23 bit/min, approaching 35 bit/min for some subjects.

Special attention was devoted to examining the effects of blinking and eye movement on the EEG pattern classification quality. Imagining of different pictures may lead to different patterns of eye movements, e.g. involuntary “scanning” of imagined picture details. Thus, contribution of EOG artifacts in EEG could facilitate EEG-based discrimination of imaginary pictures. To demonstrate the efficacy of BCI based on eye movement and blinking recognition, a designated session was conducted for each subject, where the subjects created 5 various patterns of EOG artifacts. Since EPOC headset provides no means to record electro-oculogram BrainProducts ActiCap was used. Recognition quality for such artifacts is significantly higher (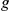 averaged 0.72) than classification quality for EEG patterns related to imagining pictures (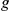 averaged 0.17). Furthermore, recognition quality of EOG induced patterns is quite high even when the EOG artifacts are suppressed on EOG electrodes by a factor more than 30. This likely caused by the remaining input from brain centers which activity is associated with eye movements (e.g. lambda waves which accompany saccadic eye movements, [41]). We demonstrate that the EEG pattern classification quality for imagining the pictures is not altered by EOG artifact removal, indicating that this recognition is based on brain centers' activity. We believe that other factors such as eye muscle activity or frowns does not impact BCI performance because frequency band optimal for classification was found to be 8–12 Hz and the half of the most relevant electrodes were posterior.

Our study is the first step in BCI research based on generation of visual images. We believe that performance of the BCI can be considerably improved. Our results are in line with this suggestion. It was shown that subjects' training enhanced the classification quality. An increase in number of electrodes also enhanced it (Figure 10). The classifiers used in this study were based only on analysis of covariance matrices. Therefore these methods ignore the frequency structure of the EEG signal although it is known that taking it into account can significantly increase classification quality (for example, [30]). The significance of frequency structure for BCI performance is also shown in the present paper. We expect that extraction and detailed investigation of signal features, increasing training time and improvements in training procedure could result in increases in both the number of recognizable mental states and the classification quality.

This study demonstrates that a relatively simple and computationally inexpensive Bayesian classifier is competitive with the classifier based on MCSP and SVM methods considered to be the most effective in BCI [28], [30]. This observation makes the method worthy of further attention. In the future work we also plan to evaluate the feasibility of the filter bank CSP method [42] for this problem.

Quite substantial quality of EEG pattern classification achieved for EPOC data and no influence of EOG artifacts on BCI performance revealed that conducting large-scale experiments in the future can be feasible. Emotiv headset usage in BCI applications is rapidly expanding. It may allow collecting large database of EEG profiles related to picture imagining.

In addition to a variety of consumer applications, BCI can facilitate the solution of a fundamental problem concerning localization of brain centers activated during imagination. Recently, functional MRI studies have supplied significant insight into this area of investigation ([11], [20], [21], [22], [23]), but these methods lack reasonable temporal resolution. We can expect that use of biological feedback which allows the subject to control how corresponding brain centers work, will ensure the stability of their activity. This will simplify localization of the active centers by solving the inverse EEG problem using any of the relevant methods [43]. At the same time, discovery of such centers will provide a clue to classification method improvement. In addition, approaches similar to described in [44] can potentially reduce time required for training and, therefore, improve image-based BCI usability.
